# A Low Mortality, High Morbidity Reduced Intensity Status Epilepticus (RISE) Model of Epilepsy and Epileptogenesis in the Rat

**DOI:** 10.1371/journal.pone.0147265

**Published:** 2016-02-24

**Authors:** Tamara Modebadze, Nicola H. Morgan, Isabelle A. A. Pérès, Rebecca D. Hadid, Naoki Amada, Charlotte Hill, Claire Williams, Ian M. Stanford, Christopher M. Morris, Roland S. G. Jones, Benjamin J. Whalley, Gavin L. Woodhall

**Affiliations:** 1 Aston Brain Centre, Aston University, School of Life and Health Sciences, Birmingham, United Kingdom; 2 School of Pharmacy, Hopkins Life Sciences Building, University of Reading, Whiteknights, Reading, Berkshire, United Kingdom; 3 Department of Pharmacology, University of Bath, Claverton Down, Bath, United Kingdom; 4 Medical Toxicology Centre, Newcastle University, Wolfson Building, Claremont Place, Newcastle upon Tyne, United Kingdom; University of Modena and Reggio Emilia, ITALY

## Abstract

Animal models of acquired epilepsies aim to provide researchers with tools for use in understanding the processes underlying the acquisition, development and establishment of the disorder. Typically, following a systemic or local insult, vulnerable brain regions undergo a process leading to the development, over time, of spontaneous recurrent seizures. Many such models make use of a period of intense seizure activity or *status epilepticus*, and this may be associated with high mortality and/or global damage to large areas of the brain. These undesirable elements have driven improvements in the design of chronic epilepsy models, for example the lithium-pilocarpine epileptogenesis model. Here, we present an optimised model of chronic epilepsy that reduces mortality to 1% whilst retaining features of high epileptogenicity and development of spontaneous seizures. Using local field potential recordings from hippocampus *in vitro* as a probe, we show that the model does not result in significant loss of neuronal network function in area CA3 and, instead, subtle alterations in network dynamics appear during a process of epileptogenesis, which eventually leads to a chronic seizure state. The model’s features of very low mortality and high morbidity in the absence of global neuronal damage offer the chance to explore the processes underlying epileptogenesis in detail, in a population of animals not defined by their resistance to seizures, whilst acknowledging and being driven by the 3Rs (Replacement, Refinement and Reduction of animal use in scientific procedures) principles.

## Introduction

Animal models of the epilepsies aim to provide researchers with a tool for use in understanding the processes underlying the acquisition, development and establishment of epilepsy, and its treatment in man. Typically, an insult is applied to the brain either via direct stimulation (e.g. kindling [1]), focal application of a toxin (e.g. tetanus [2]) or systemic administration of a proconvulsant agent (e.g. kainic acid [3], pilocarpine [4], or pentylenetetrazole [5]. In this regard, kindling or lesioning approaches could be considered more subtle than systemic drug administration, however, they may also fail to reflect the natural history of epileptogenesis, since an *a priori* choice must be made about which brain region or structure to kindle or lesion and spontaneous recurrent seizures are not always be manifest. Similarly, the repetitive nature of stimulation through kindling might be argued to be less representative of human seizure disorders where often a single insult (e.g. head trauma) is the precipitating factor. By contrast, systemic drug administration to elicit global seizure activity allows a ‘self-selection’ of brain regions most vulnerable to seizure, although, again, any advantages this brings are also subject to compromise. For example, some systemic protocols may involve such intense and/or prolonged seizure activity that neuronal damage is not localized, and again the model may fail to mimic the natural history of epilepsy in man. In this regard, Sloviter [6] has questioned the validity of epilepsy models that rely upon prolonged *status epilepticus* (SE) for the induction of the disease.

The recent adoption of 3Rs (Replacement, Refinement and Reduction of animals) principles into the research ethics framework of many EU and non-EU nations has provided an impetus to seek more refined animal models that not only reduce animal usage and suffering but also aim to improve the scientific basis for models through, for example, closer homology to the natural history of a disease. With these aims in mind, we have developed a chronic model of epilepsy and epileptogenesis, based upon an original low-dose pilocarpine model first reported by Glien et al., [7]. The Reduced Intensity Status Epilepticus (RISE) model we have developed has very low mortality (1%) but retains high epileptogenic morbidity. It also shows features consistent with epileptogenesis in man, including a lack of gross damage to the brain, a progressive profile of network alteration within the temporal lobe, with electrophysiological features similar to those seen in *in vitro* recordings from human brain tissue, and culminates in spontaneous recurrent seizures (SRS). The model is also readily repeatable across sites and hence easy to adopt. Here, we describe the induction process, the characteristics of the acquisition process, and a simple way to monitor the development of SRS during the epileptogenic latent period. In addition, we have used our novel model to examine dynamic network changes in hippocampal CA3 at selected time points following the induction process, showing that destruction of CA3 is not required to generate spontaneous seizures. Since establishing an optimal protocol for RISE, a total of 396 rats in 43 cohorts have undergone seizure induction. During these procedures, 12 animals died during the initial acute SE and 49 animals were either killed for health and welfare reasons (39) or for preparation of acute brain slices (10). In the last 20 cohorts totalling 196 animals across two institutions following the full establishment of the model, mortality occurred in only 2 animals. This translates to an associated mortality rate of ~1%, representing a significant refinement when compared to either a standard Li-pilocarpine model or the unrefined high dose pilocarpine model where mortalities are typically 40–50% [7] [8] and 50–80%, respectively [9]. Amongst the surviving animals using the RISE model, monitoring the manifestation of SRS revealed that 40–100% of animals in any cohort (mean 69% overall), monitored for 12 weeks after induction, successfully developed SRS.

## Materials and Methods

### Ethics statement: Rat electrophysiology

All procedures on rats (196 animals) were reviewed and agreed with the Bioethics Committees at Aston University and University of Reading, and were compliant with current UK Home Office guidelines and project and personal licences. Animal suffering during epileptogenesis was markedly reduced (and is the subject of this manuscript) through interventions designed to reduce seizure intensity and post-seizure complications. All experiments and procedures were performed in compliance with the ARRIVE guidelines as laid out by the UK National Centre for Replacement, Refinement, and Reduction of animals in Research (NC3Rs), who funded this study. A total of 396 rats in 43 cohorts underwent the induction procedure across the two sites.

### Ethics statement: Human brain tissue electrophysiology

Human tissue from paediatric patients undergoing surgery for drug-refractory seizures was obtained via collaboration with Birmingham Children's Hospital and with the approval of, and according to the terms specified by, both the Black Country LREC (protocol 10/H1202/23) and the Birmingham Children's Hospital NHS Trust (RECREF 10/H1202/23). Informed consent was obtained from patients and/or next of kin and recorded in a site file, and this procedure was approved by Aston University and Birmingham Children’s Hospital Trust ethics committees. Briefly, surgically removed tissue from locations predetermined using intraoperative and/or implanted ECoG was placed in cooled sucrose based artificial cerebral spinal fluid (sACSF) and slices prepared as described below. Following recovery for at least 60 minutes in an interface holding chamber, slices were transferred to an interface recording chamber and spontaneous activity recorded using the local field potential (LFP) approach described below.

### Animals

The chronic model of epilepsy developed here was based on a modified version of the lithium-low dose pilocarpine model originally described in rats [7]. This model employed a sensitizing pre-dose of lithium followed, after 24 hours, by repeated (30 min intervals) low-doses of pilocarpine. We have adopted a similar protocol but combined this with administration of the sedative and muscle relaxant drug, xylazine, during the acute seizure phase, in addition to rapid curtailment of the acute seizure phase with an anticonvulsant/antiepileptic cocktail.

Rats (male Wistar, 45-80g) were housed in temperature and humidity controlled conditions with a 12/12h light/dark cycle and were allowed to feed and drink *ad libitum*. All procedures were approved by Aston University’s local bioethics Committee and that of the University of Reading. On day 1, rats were treated with lithium chloride (LiCl, 127 mg/kg) via subcutaneous (SC) injection. Twenty-four hours following the LiCl, α-methyl scopolamine 1mg/kg (SC) was administered to reduce peripheral manifestations of muscarinic cholinergic receptor activation. Thirty minutes later, a low dose of pilocarpine (25 mg/kg, SC) was given. Animals were then closely observed for signs of seizure activity, and seizure severity was ranked using Racine’s scale (Racine, 1972). Animals failing to reach Racine Stage 4 after the first injection were dosed again with pilocarpine, up to a maximum of 3 doses (25–75 mg/kg each) at 30–45 minute intervals.

When a seizure severity rating of >3 on Racine’s scale was reached (bilateral forelimb clonus with rearing), xylazine (2.5 mg/kg intramuscularly—IM) was immediately administered. To reduce seizure severity, rats were allowed to remain in xylazine-modified SE for no more than one hour, at which point seizure activity was arrested using a ‘stop’ solution, given SC at 1 ml/Kg, and containing MK-801 (0.1 mg/kg; R&D systems, UK), diazepam (2.5 mg/kg; ethanolic solution; Bayer, De) and MPEP (20 mg/kg; Abcam Biochemicals, UK). Behavioural signs of SE ceased within 30 minutes and animals were then closely monitored during subsequent recovery. During the recovery period, rats were kept on a heat pad to maintain body temperature. They were visually monitored until ambulatory and able to consume water and moistened, powdered food. Where necessary, 0.9% saline was given intraperitoneally (IP) or SC to rehydrate animals. In most cases, recovery was nearly complete at 4 hours, and all rats were fully recovered within 12 hours. Regular checks were made to ensure animals were recovering well, and animals were weighed at 24h and 48h to additionally monitor recovery. The end-point for weight loss was 20% of weight at induction, and this was not reached at any point. Hypromellose eye drops were used during SE to prevent subsequent ocular keratitis. At approximately 48–72 hours after recovery, rats were housed in groups of 2–5 and allowed to feed and drink *ad libitum*.

For experimental purposes, comparison of network activity was conducted between epileptic rats and age matched control rats (often litter mates) that had received no pharmacological treatments.

### Behavioral monitoring for assessment of seizure development and frequency

To develop a method of assessing SRS development, two weeks after the acute SE and during the epileptogenic latent period 8 animals were individually housed and monitored for manifestation of SRS using closed-circuit television cameras (TP-101BK, Topica, Taiwan) coupled to Bluecherry video acquisition software (Bluecherry, Fulton, USA [9]) for 24 hours a day for up to 3 weeks. Here, animals also concurrently underwent behavioural testing using the post-seizure behavioural battery (PSBB; see next section) and test scores were correlated with the presence or absence of SRS.

In a separate experiment, ten epileptic animals were video monitored for 3 weeks for 12 hours per day (19:30–7:30) to assess severity and frequency of seizures following the appearance of SRS. Video monitoring was conducted during the white light period, as ~65% of spontaneous seizures in this type of model occur during this time [10]. Animals were considered to exhibit SRS when video evidence revealed a seizure severity score ≥3 (bilateral forelimb clonus) on Racine’s scale [11]. SRS were monitored for varying durations depending on the experiments, ranging from a few weeks to several months.

### Post-Seizure Behavioural Battery

Animals that have undergone an induction protocol using the original pilocarpine or Li-pilocarpine model show behavioural modifications characterized by hyperexcitability and aggression when compared to normal animals [12] [13] [14]. To examine whether such behavioural alterations are also present in our refined model, we employed a modified version of the behavioural battery developed by Moser et al. [9] and adapted by Rice et al. [13] to assess these changes. The following two simple and non-stressful tasks constituted the PSBB.

#### Touch task

The animal is gently prodded in the rump with a blunt instrument (e.g. scalpel handle). Responses are scored: 1, no reaction; 2, rat turns toward instrument; 3, rat moves away from instrument; 4, rat freezes; 5, rat turns toward the touch; 6, rat turns away from the touch; 7, rat jumps (with or without vocalisation).

#### Pickup task

The animal is picked up by grasping around the body. Responses are scored: 1, very easy pickup; 2, easy pickup with vocalisation; 3, some difficulty in pickup (rat rears and faces the hand); 4, rat freezes; 5, difficult pickup (rat moves away); 6, very difficult pickup (rat behaves defensively or attacks the hand).

PSBB tasks were administered twice weekly, starting two weeks after SE induction and continued for 10 weeks in individually housed animals. Behavioural responses to touch and pickup tasks were evaluated by trained experimenters and compared to age-matched control (non-epileptic) animals. Concurrent video recordings during the latent period (see above) were used to monitor the development of SRS and initially validate application of the PSBB to distinguish between known epileptic (n = 6, data not shown), non-epileptic SE-induced (n = 2, data not shown) and non-epileptic control animals (n = 12). In order to further validate its use to identify animals exhibiting SRS, a larger cohort of animals having undergone *status epilepticus* induction but without video confirmation of SRS (n = 37) were tested. No animal exhibited a seizure event during administration of the PSBB task.

PSBB scores were analysed by calculating the product of the touch and pickup scores (‘touch x pickup’) and the average pickup score over four consecutive tests (‘time-bin pickup’). A repeated-measures 2-way ANOVA was used to assess differences between PSBB scores for different cohorts. A non-parametric Spearman's rank correlation was carried out to assess if a relationship could be observed between pilocarpine dose administered during induction and the week animals were confirmed epileptic by PSBB or during handling.

### Slice preparation and electrophysiology

Rats were anaesthetized using isoflurane followed, after loss of consciousness, by pentobarbital (60 mg/kg, SC) and ketamine/xylazine (100 and 10 mg/kg respectively, IM). Transcardial perfusion was then performed using ice-cold sACSF. Animals were decapitated and the brain removed and placed in ice-cold sACSF. Combined entorhinal-hippocampal slices were cut using a vibratome (Campden Instruments, UK) to a nominal thickness of 450 μm, as described previously (Jones and Heinemann, 1988). Slices for extracellular recordings were stored in an interface chamber (or in an interface holding chamber (Harvard, UK)) containing normal ACSF bubbled with carbogen (95%O_2_/5%CO_2_). Slices were allowed to recover in the holding chambers for a minimum of 1 hour. The sACSF was composed of (in mM): sucrose (151), KCl (2.5), MgCl_2_ (10), NaHCO_3_ (25), Na_2_HPO_4_ (1.25), CaCl_2_ (0.5), d-glucose (10), ascorbic acid (1). The ACSF used for recording had the following composition (in mM): NaCl (126), KCl (2.5), MgCl_2_ (1), CaCl2 (2.5), NaHCO_3_ (26), NaH_2_PO_4_ (2), d-glucose (10). Both had a pH of 7.4 and an osmolarity of 310 mOsm. The sACSF solution used for cutting, (but not for storage) also contained N-acetyl cysteine (2), taurine (1), indomethacin (0.045), uric acid (0.3), ethyl pyruvate (20) and aminoguanidine (0.2), a combination of neuroprotectants that we (Woodhall, G.L. and Jones, R.S.G., unpublished observations) have found to prevent excitotoxic damage, reduce cell death, and enhance slice longevity without apparent effect on the pharmacology of glutamate or GABA transmission.

### Extracellular Recording

Slices were transferred to an interface recording chamber where they were maintained at the interface between a continuous perfusion of oxygenated ACSF (1–2 ml/min) maintained at 32 ± 0.5°C and gassed with warm, moist carbogen. Slices were viewed using an Olympus ZX51 dissecting microscope for visual placement of 3–4 recording electrodes at selected sites. Electrodes were pulled from filamented borosilicate glass (BF120-69-10, Intracel, UK), using a Sutter P-97 electrode puller (Intracel, UK) and had resistances of 8–10 MΩ after filling with ACSF. Simultaneous recordings were made from layers II and V of the medial entorhinal cortex (mEC) and CA3 and CA1 of the hippocampus. Network activity was recorded extracellularly using an EXT-02F amplifier (NPI—Scientifica, UK). Signals were amplified at an overall gain of 1000 and band-pass filtered at 0.1–1.3 KHz. Data were analysed offline using Spike 7 (CED, Cambridge, UK) and Matlab software (R2012b, Mathworks, USA). Power spectra were determined for 30 s epochs of data using the FFT algorithm in Spike 7, and peak power and frequency were compared. One-dimensional Morlet continuous wavelet analysis (cwt) was performed in Matlab using 1.6 s epochs of data downsampled from 10 kHz to 1 kHz, with a step value of 1 and maximum scale of 128 or 256. Pseudo-frequency values as related to scale values were determined for each plot and a dashed box corresponding to the 20–60 Hz range added to each wavelet spectrogram. Spontaneous ictal-like synchronized events were identified by eye, and as far as possible within the limits of point recordings of activity, the seizure onset site and progression path within the combined slices were determined.

## Results

### RISE induces SRS with high morbidity

**Fig 1:**
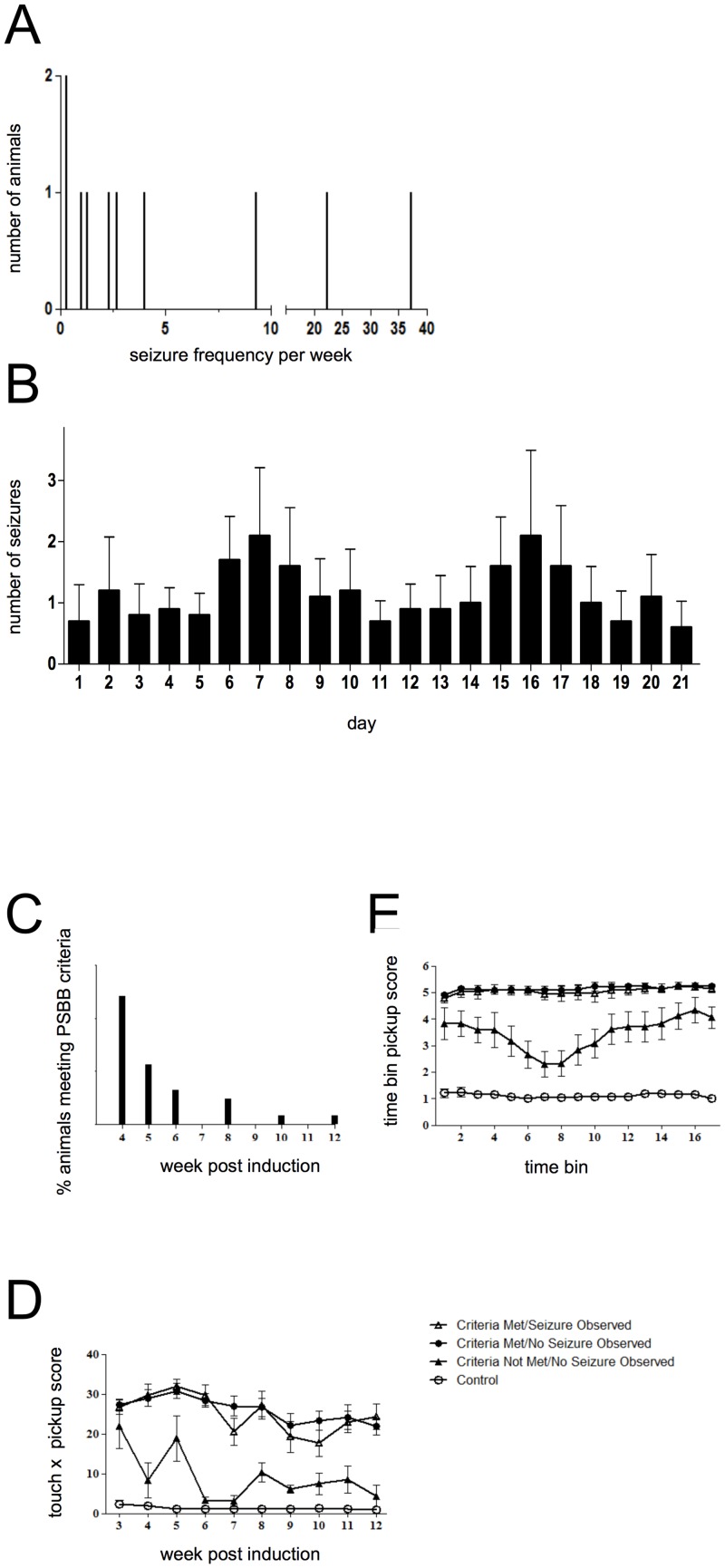
Behavioral measurements of epilepsy following induction. **A.** Distribution of weekly seizure frequency as observed during video monitoring (n = 10) **B.** Mean daily seizure frequency of animals during the recording period, data are expressed as mean ± SEM (n = 10). **C.** The percentage of animals meeting PSBB criteria at various weeks post-induction (n = 31). **D.** Touch x pickup and **E.** time bin pickup PSBB scores of animals having undergone *status* epilepticus induction (n = 37) and age-matched controls (criteria met/seizure observed, n = 13; criteria met/no seizure observed, n = 18; criteria not met/no seizure observed, n = 6; control, n = 12).

The course of development of SRS in the unrefined high dose pilocarpine model has been described previously in detail [15] [16]. Here, we describe the comparative situation for the highly refined model with very low mortality. Thus, following confirmation of SRS (with PBSS and/or visual confirmation), seizure characteristics were examined in behaviorally monitored animals (see below for details, n = 10). Animals varied greatly in the incidence of seizures (range: 1–37; mean: 8.1 ± 3.5 seizures per week; (Fig 1A). Despite the large inter-animal variation in seizure frequency, a characteristically periodic variation was also observed (Fig 1B) with clear peaks every 5–7 days. Interestingly, these patterns could not be attributed to external factors (e.g. periods of specific activities by personnel in the animal housing facility, light/dark periods, bedding changes) as video monitoring schedules differed between animals since they were not only recruited from a number of different inductions but also began to exhibit SRS at different times.

### RISE elicits SRS that can be detected using PSBB tasks

A significant issue associated with models of SRS is the unpredictability of onset and detection of seizure events, which necessitates continuous video monitoring and laborious replay and assessment. Moreover, the time required to review video records can mean animals are not confirmed as epileptic until days or weeks after their first seizure, which hinders effective use of the animals for studies of epileptogenesis and epilepsy e.g. by delaying initiation of drug treatments at a clinically pertinent time point. An alternative approach is to implant electrodes to enable continuous monitoring of ECoG for seizure activity. Again, this is labour and equipment intensive to establish, time consuming to analyse and increases animal use since ECoG implantation in animals prone to convulsive seizures are prone to detachment. Thus, we have attempted to establish a simple behavioral paradigm to verify that SRS are induced by RISE and to identify the point at which SRS develop. Accordingly, we have validated the use of PSBB for this purpose.

Initially, animals (n = 8) were video-monitored during the latent phase to assess the development of SRS. Concurrently, we conducted PSBB testing in these 8 animals, of which 6 exhibited SRS. The epileptic animals consistently exhibited high PSBB scores for both tasks (touch x pickup: 12.6 ± 1.4; pickup time bin: 4.2 ± 0.2; n = 6), while age-matched non-epileptic controls reliably exhibited low PSBB scores (touch x pickup: 1.3 ± 0.1; pickup time bin: 1.1 ± 0.0; n = 12). Animals that underwent induction but did not develop SRS had variable and higher PSBB scores than controls for both tasks, although these were lower than in epileptic animals (touch x pickup: 5.2 ± 0.8; pickup time bin: 3.4 ± 0.3). Using the differences between these scores we developed the following criteria to identify animals that exhibit SRS: touch x pickup score is >10 or time-bin pickup score is ≥4 for 4 consecutive trials. Of the 6 animals that exhibited SRS, 83% fulfilled the criteria although neither the unconfirmed, non-epileptic animals (n = 2) nor the age-matched non-epileptic controls did so, demonstrating the stringency of the chosen criteria, which permit limited false negatives but not false positives.

Having established a reliable means of identifying SRS manifestation, we next examined the duration and variability of the latent period; viable models of epileptogenesis and epilepsy require this period to be long enough to reflect the human condition but short enough to enable practical study. Particularly aggressive models, such as the unrefined high dose pilocarpine model, result in faster manifestations of SRS (14 ± 11 days; [8]). However, these result in significant neurodegeneration in numerous brain regions, in particular the hippocampus and piriform cortex, making these models less representative of human TLE [17]. Here, we used PSBB to monitor 37 animals that underwent SE induction, of which 84% met the SRS criteria within 12 weeks of induction and were used in this analysis; the majority met the criteria 4 (48%) or 5 weeks (23%) after induction (Fig 1C).

The PSBB scores of these animals and age-matched control animals (n = 12) were then categorized on fulfilment, or not, of the criteria and the presence, or not, of visually confirmed seizures (Fig 1D and 1E). All animals that met the criteria received significantly higher (*P*<0.001) touch x pickup and pickup by time bin scores (Fig 1D and 1E) when compared to age-matched controls. Although there was no significant difference (*P*>0.05) between scores received by induced animals that did not meet criteria and those of age-matched controls, the scores of the former exhibited considerable variability, which may reflect slower development of an epileptic phenotype i.e. >12 weeks. Animals not meeting the criteria were never seen to have seizures. For animals that did meet the criteria, touch x pickup and pickup by time bin scores were consistently high and no statistically significant difference was found between animals that exhibited SRS and those that did not. This validates the use of PSBB alone to identify epileptic animals in this model without requiring visual verification of SRS. No significant correlation between pilocarpine dose administered during SE induction and the week of SRS manifestation was found (*P*>0.05, n = 38).

### Networks changes during epileptogenesis in the RISE model

Following a period of RISE not exceeding 60 minutes, we randomly assigned animals to groups and made recordings at 24 hours, 5–6 weeks and 90+ days post-induction. At these time points following induction, we made horizontal brain slices containing both hippocampus and entorhinal cortex [18] cut such that connections around the extended circuitry remained intact. Recordings were made in hippocampal area CA3 at the indicated time-points in order to track the progression of epileptogenesis and to monitor the functional state of neuronal networks in CA3. During the post-insult development of SRS, we noted clear differences between the activities of neuronal networks relevant to temporal lobe epilepsy.

**Fig 2:**
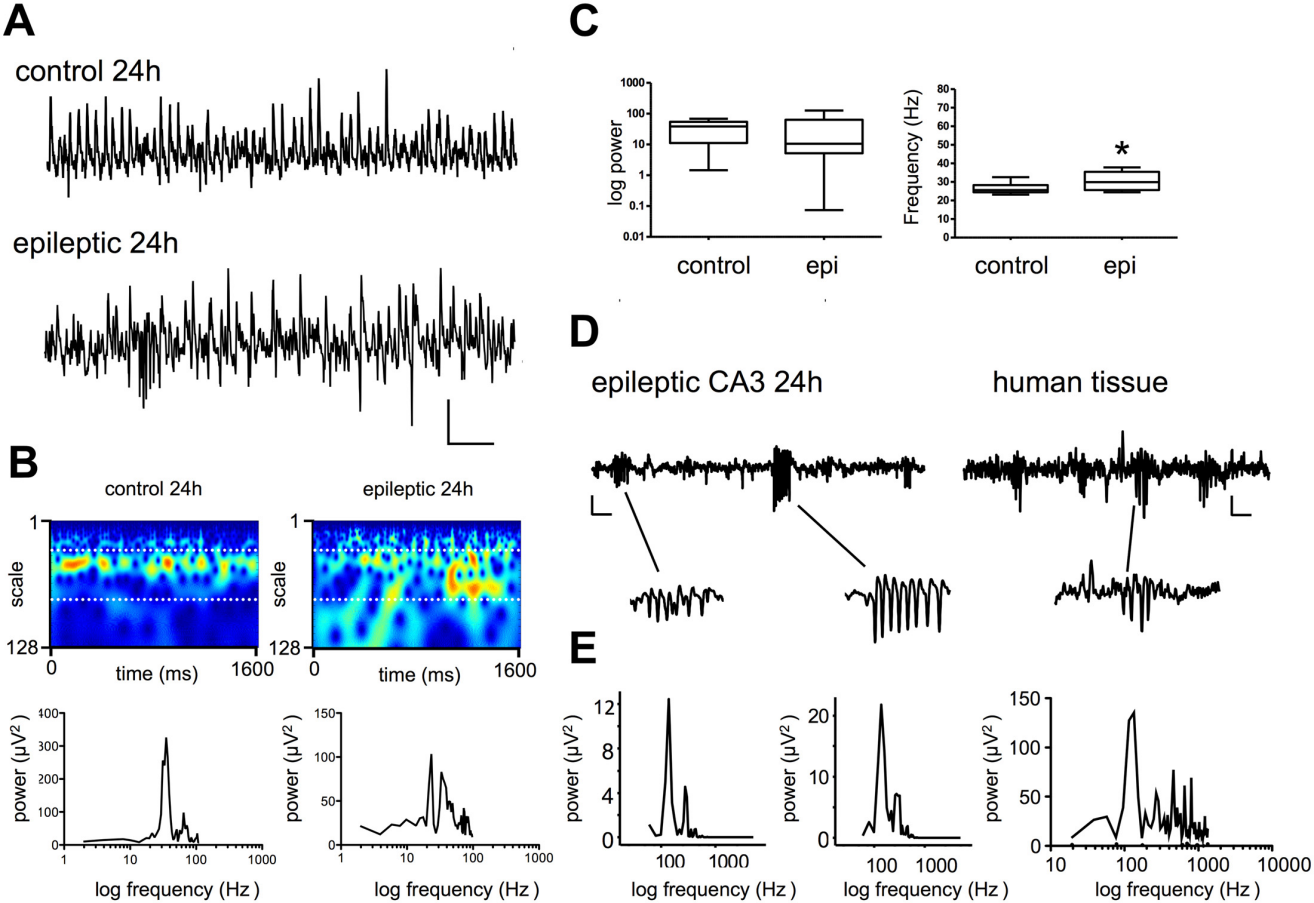
At 24h post induction LFP recordings show gamma oscillations and fast bursts in CA3. **A.** Example traces recorded 24h post induction and in controls. Scale bar 200 ms x 50 μV. **B.** Example Morlet-wavelet spectra and power spectral density plots of the same traces, white dotted lines on spectra indicate 20–60 Hz pseudo-frequency band. **C.** Pooled power and frequency plots for control and PSE 24. **D.** Fast ripples recorded in CA3 at 24h and similar activity seen in temporal neocortex from a human patient with TLE. **E.** Power spectra from ripples identified in D (rightmost panel from human tissue).

**Fig 3:**
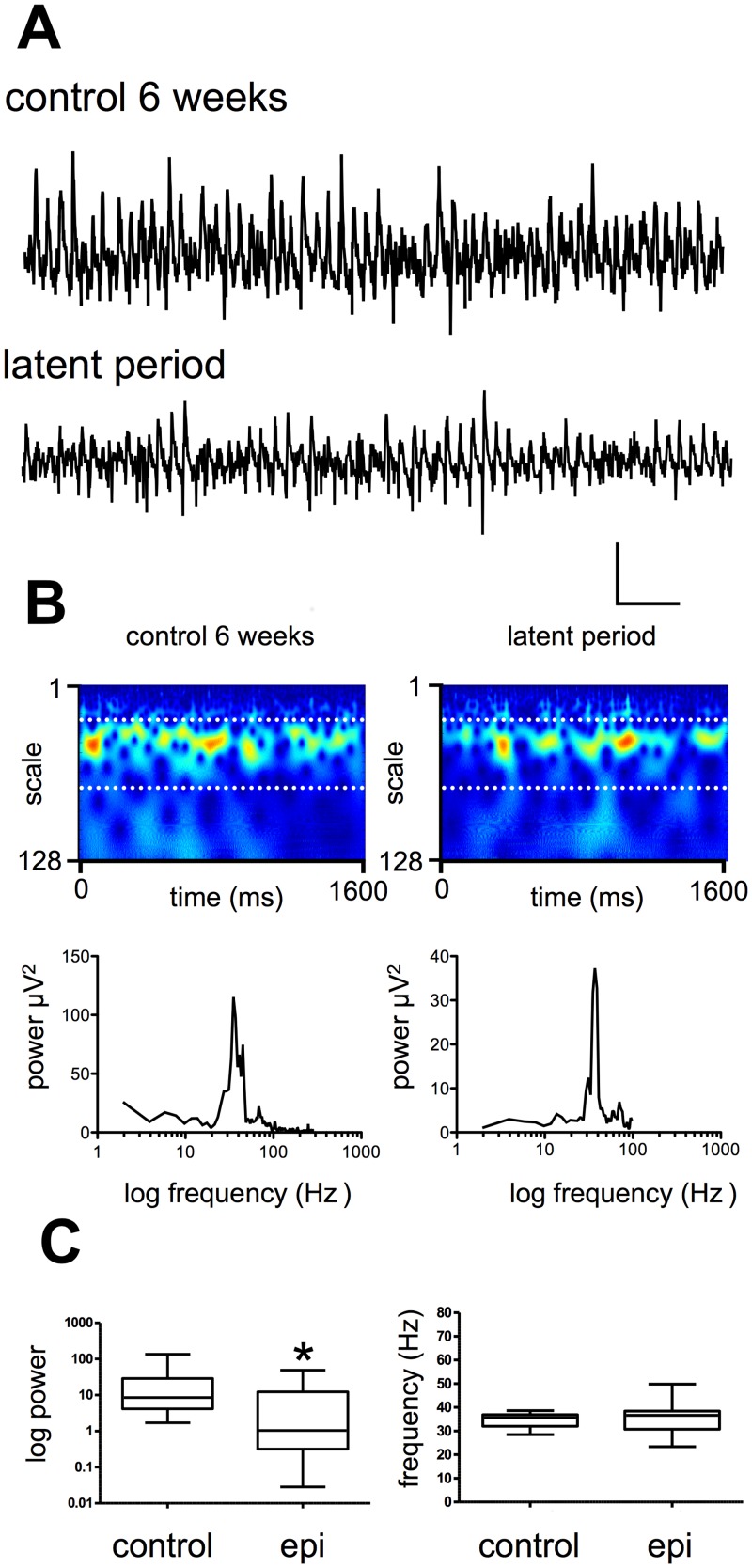
Spontaneous network activity in CA3 during the latent period. **A.** Raw data from CA3 recorded during the latent period and in controls. Scale bar 200 ms x 50 μV. **B.** Power spectral density plots of the activity shown in A. **C.** Example Morlet-wavelet spectra and power spectral density plots of the same traces, white dotted lines on spectra indicate 20–60 Hz pseudo-frequency band. **C.** Pooled power and frequency plots for control and latent period.

In experiments in drug-free slices from age-matched control animals, we routinely observed spontaneous beta/gamma (hereafter referred to as gamma) activity in CA3 (mean peak frequency 28.5 ± 1.2 Hz, mean peak power 34.7 ± 10.2 μV^2^ n = 6). Fig 2A shows a typical recording from CA3. At 24h post-induction (PSE 24) spontaneous activity at gamma frequency was also routinely observed (mean frequency 30.5 ± 1.1 Hz, mean peak power 36.6 ± 10 μV2, n = 18) and bursts of fast oscillations (range 136–670 Hz, n = 13) were notable at intervals ranging from 0.6–8.8 s. Typical data are shown in Fig 3A. Comparisons of power spectra and wavelet spectrograms and those for control and PSE 24h (Fig 2B) show a similar profile of gamma oscillatory activity under both conditions. However, a complex mix of low and high frequency oscillatory activity associated with spontaneous gamma oscillations was characteristic of CA3 at PSE 24 and this is exemplified in Fig 2B. In pooled data from 6 control and 18 PSE 24h recordings (Fig 2C) there was no statistical difference in mean peak power (*P* >0.05, Kruskall-Wallis test) between the two groups, however, the slight difference in mean peak frequency (reported above) was statistically significant (*P* <0.017, Kruskall-Wallis test). In order to determine the stability of periodic spontaneous network activity over time, we computed the autocorrelation function for short (1.6 s) epochs of gamma activity in CA3. Under control conditions, the mean peak autocorrelation function was 0.23 ± 0.04, and this was significantly higher than that determined at PSE 24 (0.15 ± 0.04; *P* < 0.04, t-test, n = 20). In a parallel series of experiments, we investigated spontaneous activity in slices of temporal neocortex taken during surgical resection to control intractable epilepsy in a paediatric epilepsy patient. Though spontaneous network gamma oscillations were not observed, epochs of fast oscillations were seen, and these showed similarities with those observed in rat CA3 (Fig 2D and 2E), suggesting that the model shows at least superficial similarity to tissue taken from epileptic brain in man.

Overall, these data suggest that at the shortest post-insult interval, activity in CA3 was altered compared to controls, with periodic bursts of fast oscillations and less stable gamma activity. However, the inhibition required to generate coherent, spontaneous network gamma activity remained generally intact. Furthermore, fast oscillatory activity in CA3 showed a profile that was largely consistent with activity observed in human tissue taken from a patient with medically intractable seizures.

In parallel to the experiments above, we performed similar investigations at PSE 1 week and during the latent period. As described above, the length of the latent period was variable between animals, and some variance was also noted between laboratories, such that rats in Reading became epileptic at typically around 4 weeks, compared to 6–8 weeks in Birmingham. In both cases, however, the majority of rats showed SRS at 12 weeks, as assessed by PSSB and/or behavioral monitoring.

We next examined CA3 activity *in vitro* at 5–6 weeks during the latent period. As shown in Fig 3, spontaneous oscillatory activity remained intact. Fig 3A shows typical recordings from slices in from age-matched control and animals 6 weeks at PSE. Spontaneous oscillations were readily observed under both conditions, with mean peak power during the latent period of 12.5 ± 6.2 μV^2^ (n = 13), as compared to control values of 25.6 ± 10.7 μV^2^, (n = 13). Statistical analysis revealed that the difference in mean peak power of spontaneous gamma activity was significantly lower in the epileptic cohort when compared to controls (Fig 3C *P*<0.007, Mann-Whitney test). Interestingly, when we analyzed the peak autocorrelation function at this time point, we found that, despite its low power, the mean value for autocorrelation of the gamma signal was significantly higher in the epileptic group compared to control (0.19 ± 0.02 vs 0.10 ± 0.02; t-test *P* <0.004, n = 14). No spontaneous epileptiform activity was seen in recordings during this period, indicating that CA3 remains functionally intact and, further, suggesting that the model does not result in grossly damage circuits in this region.

**Fig 4:**
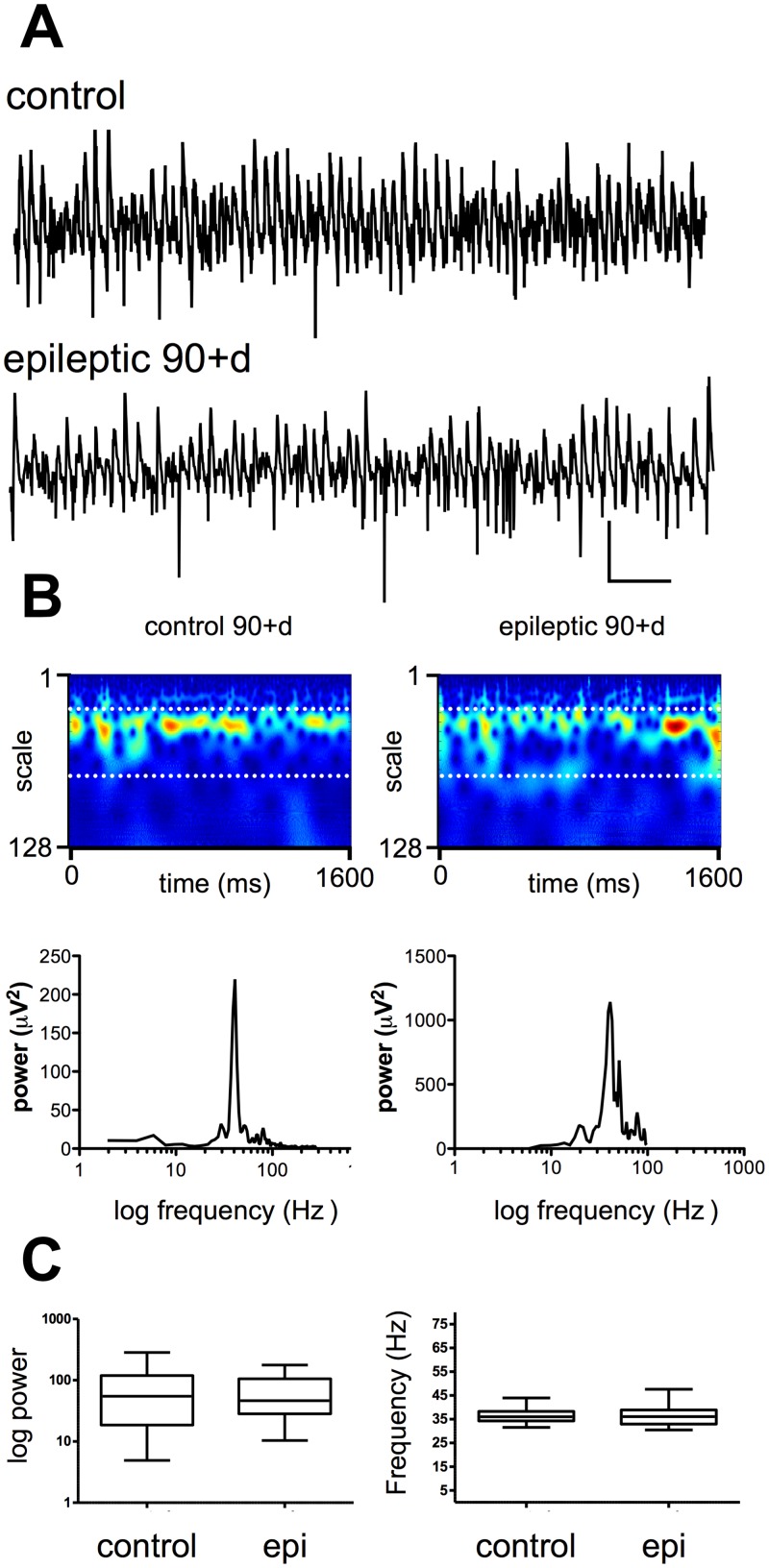
Spontaneous network activity in CA3 following establishment of SRS. **A.** Raw data from CA3 recorded 90 days post-induction and in controls. Scale bar 200 ms x 50 μV. **B.** Power spectral density plots of the activity shown in A. **C.** Example Morlet-wavelet spectra and power spectral density plots of the same traces, white dotted lines on spectra indicate 20–60 Hz pseudo-frequency band. **C.** Pooled power and frequency plots for control and PSE 90d.

**Fig 5:**
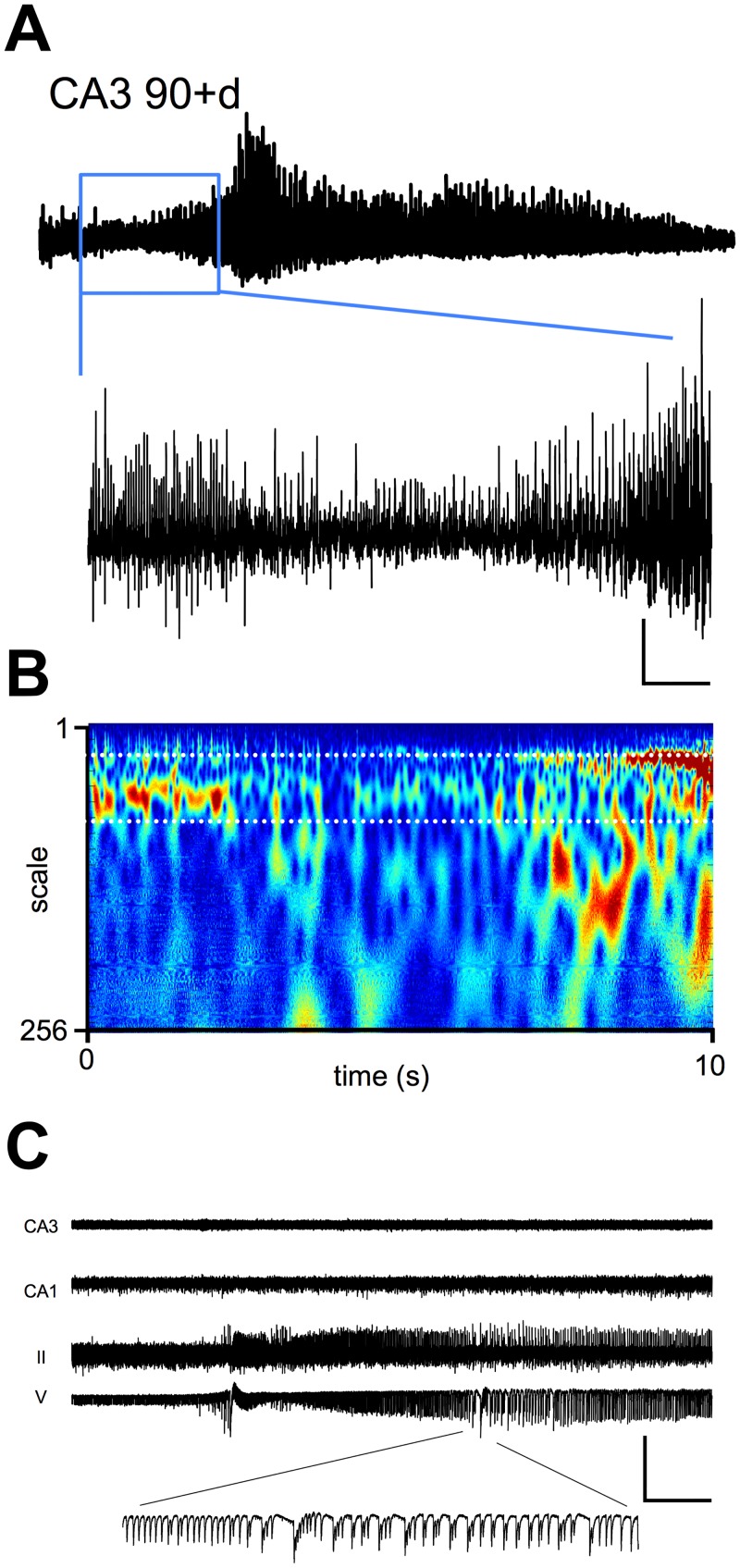
Appearance of ictal-like events in CA3 and mEC in vitro. **A.** Example ictal-like event recorded in CA3 in slices from a PSE 101 day animal with confirmed SRS. Trace length 50 s. Blue box denotes expanded timescale (scale bar 950 ms x 550 μV). **B.** Example Morlet-wavelet spectrum of the area delineated by the blue box in A. **C.** Example recording made in CA3, CA1, layers V and II of mEC during an ictal-like event generated in mEC (scale bar 950 ms x 500 μV), a section is shown at an expanded temporal scale for clarity.

Next, we examined the properties of spontaneous activity in CA3 at 90+ days PSE, when animals were showing SRS. At this time point (Fig 4A), (in slices from rats now weighing 400-500g), CA3 was still spontaneously active, generating similar gamma frequency network oscillations (mean peak power 79.1 ± 23.4 μV^2^ at 35.6 ± 0.9 Hz, n = 28) to those observed in controls (mean peak power 65.7 ± 11 μV^2^ at 36.7 ± 0.9 Hz, n = 18), again suggesting that CA3 remained functionally intact. Fig 4A shows typical spontaneous gamma activity in CA3 recorded at 90 days PSE and in age-matched control slices. The power spectra and Morlet-wavelet plots in Fig 4B show a strong similarity between activity recorded in control and epileptic animals, and, as Fig 4C shows, mean power and frequency were very similar between groups (*P*>0.98 for mean peak power; *P*>0.79 for frequency). However, during these recordings in slices made from animals manifesting spontaneous recurrent seizures, spontaneously generated ictal-like discharges could be seen arising in CA3 and in medial entorhinal cortex (mEC). Fig 5A shows such a discharge recorded at PSE day 101, and initiated in CA3. As the expanded timescale trace and Morlet wavelet spectrogram show, spontaneous oscillatory activity collapses for a short period just prior to initiation of an ictal-like discharge containing high frequency population activity.

Interestingly, when we observed ictal-like events in the first week following induction, these almost always commenced in hippocampus. Following the development of SRS, ictal-like events observed *in vitro* often appeared to be initiated in mEC. We analyzed recordings from slices at 24h, during the latent period and following development of SRS. Ictal-like events were rare in CA3 at 24 hours (3/20 recordings) and absent in mEC at this time point, and we did not observe ictal-like events during the latent period at all in more than 60 recordings. Following development of SRS, we observed ictal like events that appeared to have been initiated in mEC. Fig 5B shows one such event, probably arising in layer V of mEC and with no concurrent activity in CA3. Given the reduced nature of the slice preparation, it is not possible to make robust statements concerning the location of the primary epileptogenic zone using these observations, however, our data do suggest that the epileptogenic zone may not be stable during the process of epileptogenesis.

#### Histology

**Fig 6:**
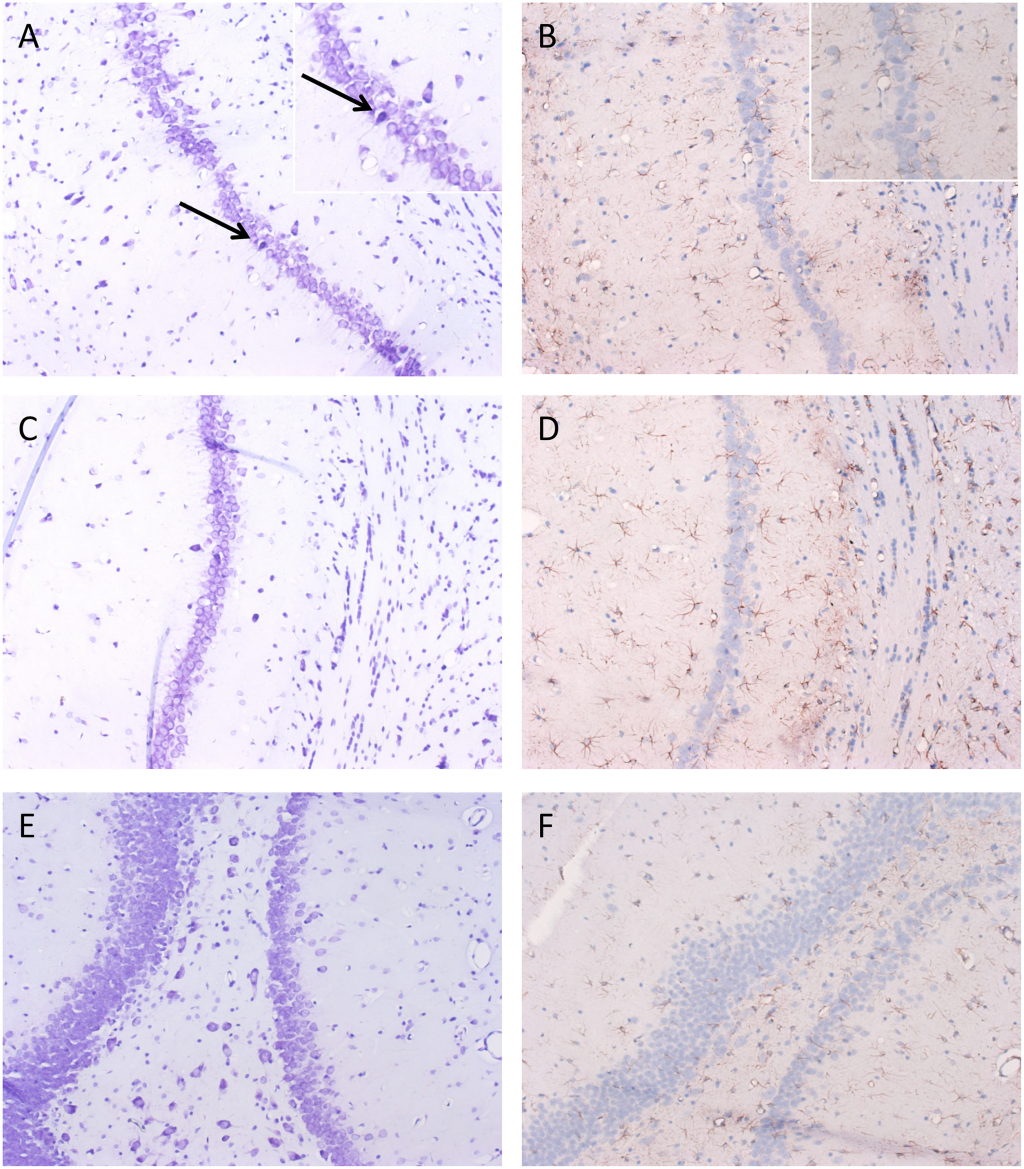
Appearance Anterior hippocampus following Pilocarpine exposure. A) CA1 pyramidal cell layer shows only slight disruption of the normal architecture of the pyramidal layer and the presence of occasional darkly stained neurones (inset higher magnification) and B) normal appearing astrocytes (inset higher magnification). This is also observed in the CA2 region with C) normal pyramidal cell layer with occasional darkly staining neurones and D) minimal astrocyte reactivity. E) The dentate gyrus and CA4 region show a normal granule cell layer, with normal appearing astrocytes (F). A-F x200 magnification, inset (B) x 200.

**Fig 7:**
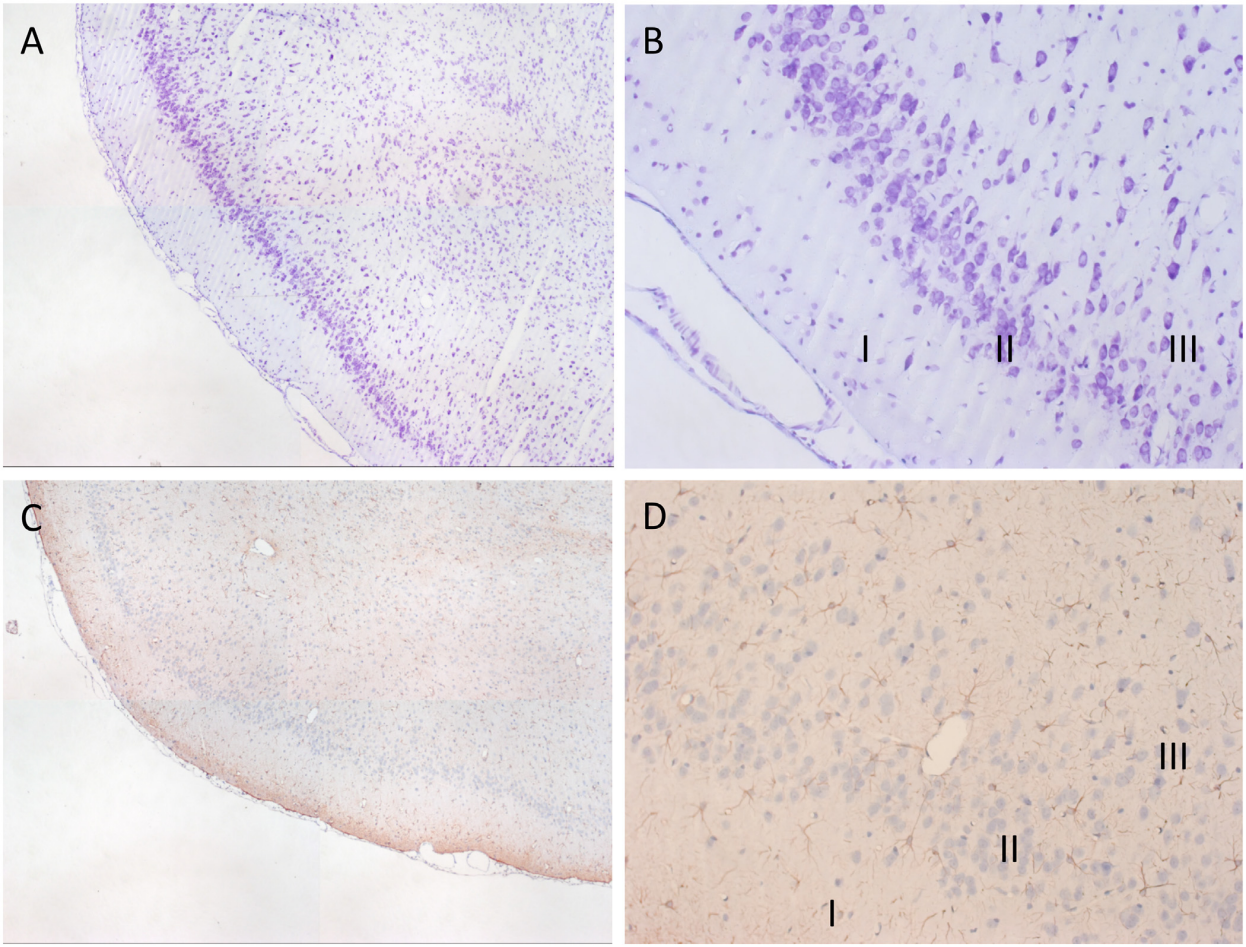
Appearance of piriform cortex following pilocarpine exposure. Normal cellular morphology of the piriform cortex showing intact layer II pyramidal cells (A,B) staining with cresyl fast violet, and (C,D) normal appearing astrocytes throughout the layers using GFAP immunohistochemistry. I, II, and III, Layers I, II, and III of piriform cortex. A,C x12.5, B,D x200 magnification.

To confirm the presence or absence of gross neuronal damage induced during the process of epileptogenesis, we conducted a histological investigation of key brain regions in a sample of animals who had experienced seizures. Following pilocarpine exposure and establishment of recurrent seizure activity, the architecture of the CA1 pyramidal cell layer showed only slight disruption and the presence of occasional darkly stained, presumably damaged neurones and normal appearing astrocytes. This was also observed in the CA2 region. The dentate gyrus and CA4 region the granule cell layer and astrocytes appear normal (Fig 6). The piriform cortex, another region associated with cell damage [8] and death during epileptogenesis, is also well-preserved in the RISE model as Fig 7 shows, with mostly normal morphology of neurones across all three pyramidal cell layers. Assessments of neuronal and glial populations were performed in laboratories in both Brimingham and Reading, and consistently found little gross neuronal damage or astrocyte reactivity in temporal lobe and piriform cortex. These data appear to confirm that the model produced limited levels of neuronal damage when compared to earlier pilocarpine-based models [19] and show that successful epileptogenesis may depend on subtle alterations in neuronal network architecture and individual neuronal behaviour.

## Discussion

The RISE model of epileptogenesis represents a highly refined model of the original low-dose Li- pilocarpine model of epileptogenesis reported by Glien at al., [7]. The model substantially reduces acute mortality resulting from uncontrollable seizure generalization, and also reduces the overall burden on animals due to the effects of prolonged seizures, with consequent reduction in later mortality. Despite a robust reduction in mortality, the RISE model retains a high degree of epileptic morbidity and exhibits features similar to those seen in epilepsy in paediatric and adult epilepsy patients, including development of fast oscillatory activity and ictal-like events both *in vivo* and *in vitro*. The periodicity of seizure frequency is similar to that previously described in the unrefined, high dose pilocarpine model, where animals showed considerable variability in these parameters [16] [20]. Moreover, this variability and clustering of seizures has also been reported in human TLE, further exemplifying the translational relevance of this model [21]. The use of behavioural tasks and stringent scoring criteria described here remove the need for tedious and labour intensive long-term monitoring of animals to reliably monitor the onset of seizures, making the study of SRS in this model more feasible and approachable for researchers.

A major innovation in the RISE model was the introduction of xylazine during early development of the acute SE. We have not directly studied the pharmacological actions of xylazine in the RISE model but it seems likely that its effects are associated with its role as a central adrenergic alpha-receptor agonist. Xylazine has previously been reported to have both pro- and anticonvulsant activity in amygdala kindling [22], with low doses enhancing kindled seizures and higher doses reducing afterdischarge (AD) threshold and duration. Joy et al., [22] suggested that xylazine was unique in “*…that it can facilitate kindling acquisition rate while simultaneously decreasing AD duration*.” We have previously reported its use in a model of epileptogenesis [23] [24]. In addition to any direct effects of xylazine on seizure threshold or ongoing activity in vulnerable neuronal networks, xylazine also affects motor activity, having sedative and muscle relaxant properties, and it seems likely that these play an important role in reduction of mortality associated with the RISE model. In behavioural terms, the most notable effects of xylazine at the dose employed here are sedation and reduction in locomotor hyperactivity and ataxia. In man, SE can be convulsive or non-convulsive and, although underlying EEG changes may be similar, the latter is associated with lower mortality (e.g. see [25]). The SE in the RISE model is obviously sufficient to elicit network changes leading to SRS, but prevention of the convulsive element may underlie the substantially decreased mortality rates. The reduced mortality through prevention of the development of running/bouncing seizures may be indicative of decreased likelihood of secondary generalization involving sub-cortical sites, particularly the brainstem [26].

A second major refinement in the RISE model was the combined administration of an mGluR5 antagonist (MPEP), the non-competitive NMDA receptor antagonist (MK-801) and diazepam to rapidly curtail the non-convulsive SE. Fujikawa [27] originally reported that another NMDA receptor antagonist, ketamine, was neuroprotective after status epilepticus. Similarly, Tang et al., [28] reported that a drug combination similar to the one used here, given intravenously, was able to halt seizures induced by a high-dose (300 mg/kg) of pilocarpine in mice, and also prevent the early hippocampal cell death elicited by the convulsant. We found that the same combination of drugs (albeit using a far lower dose of MPEP and a higher dose of diazepam) given by SC injection produced rapid termination of RISE in rats. We also saw little in the way of cell damage in hippocampal and adjacent cortical areas at early or late time points following RISE, and the ‘stop’ cocktail may be important in this. Together, the reduction of convulsive effects induced by xylazine, and the rapid termination of the SE, resulted in radically reduced mortality associated with epileptogenesis using other non-refined pilocarpine models.

Mortality in pilocarpine based models is infrequently quoted in published papers, and, in the UK, the National Centre for Replacement, Refinement and Reduction (NC3Rs) has driven the development of the ARRIVE (Animal Research: Reporting of *In Vivo* Experiments) guidelines to ensure consistency in reporting of research involving animals. Figures that are reported vary greatly but are often very high (sometimes >90%), even in low dose pilocarpine protocols (see [8]). The RISE model reduces mortality close to zero, without greatly affecting the generation of a chronic epileptic condition. In addition, the use of xylazine largely circumvents the highly stressful convulsive activity during acute SE. With respect to animal project licensing and animal welfare purposes, previous models of acquired epilepsy have always been categorized by the Home Office in the UK as being of ‘substantial’ severity due to the stressful effects of the acute induction and the associated mortality. The RISE model has now been classified as ‘moderate’ severity at Aston University. It should also be noted that the low mortality rate obviously results in fewer animals being used overall, so the RISE model clearly fulfills at least two (refinement and reduction) of the 3Rs.

High mortality in previous models seems to be related, at least in part, to the duration of the period spent in SE. Indeed, Sloviter [6] has argued that prolonged periods of SE leads to global cortical damage, inflammation and cerebrovascular lesions and that, as a consequence, the recurrent seizures generated in many models do not reflect the natural history of epilepsy in any meaningful way. By minimizing the intensity of the acute SE we essentially eliminated mortality, but were still able to record consistent epileptogenesis leading to SRS after a latent period. Histological examination of limbic brain regions and piriform cortex of epileptic animals reveals no marked signs of neuronal damage or loss. We also measured the functional state of the neuronal networks using LFP recording and found that the vulnerable CA3 region of hippocampus remained spontaneously active *in vitro* throughout the development of chronic epilepsy, strongly suggesting that our model does not result in wholesale excitotoxic damage to this region. At the same time, whilst abnormal neuronal activity can be demonstrated in CA3 as early as 24 hours after the initial insult, this abnormal activity appears to resolve during the latent period, with the area showing relatively normal spontaneous activity. Later, abnormal activity in the form of ictal-like events appears in the hippocampus and entorhinal area following the development of behavioural SRS. These data indicate that processes underlying epileptogenesis can be readily explored within the model, and suggest that the hypothesis of a stepwise process involving multiple neuronal regions in the temporal lobe (and beyond) may be involved in the development of SRS can be tested. A summary of the key network features as measured in CA3 *in vitro* and in terms of the PSBB is presented in Table 1 (below). It is interesting to note that whilst the pattern of establishment of epilepsy was similar between the two laboratories, the duration of the latent period did differ between laboratories, and this may reflect subtle differences between in-house bred rats (Aston) and those purchased from commercial suppliers (Reading). This variability does, however, indicate that the model is robust with respect to the source of rats used, and it is hoped that this contributes to the portability of the model between laboratories.

**Table 1:** Summary of *in vitro* network and behavioural observations at different stages of the RISE model.

Time point	Network activity	Behaviour/PSBB
control	Gamma band	Normal/no aggression
24h post status	Theta, gamma and HFO	Oro-facial twitches; forelimb clonus; no aggression or aversion
Latent period	Low power gamma	Normal; some animals showing aggression on handling
SRS	High power gamma	Recurrent seizures; aggression on handling; backing away from foreign objetcs

Together, the use of the low-dose lithium protocol, minimization of time spent in SE, administration of xylazine and the use of the multi-drug cocktail provide a highly reliable and replicable method for induction of epilepsy, which reduces mortality whilst maintaining a high degree of epileptogenicity. A criticism often leveled at animal models of chronic epilepsy by clinical colleagues is that patients almost never present with epilepsy that has resulted following an episode of SE, and, as such, models employing SE during induction are fundamentally unable to mimic human epilepsy (see also [6]). The current model, whilst retaining an element of short-duration, modified SE, goes some way to mitigating this criticism, and shows that prolonged SE is not necessary for the reliable induction of a condition with significant similarities to human epilepsy. In this context, it was recently reported ([29]) that animals treated with pilocarpine but that did not enter SE showed slow development of SRS, suggesting it may not be the critical factor in epileptogenesis, and indicating that further development of the current model may be possible. In this regard, refinement of the protocol for administration of lithium, which is an irritant, is both desirable and necessary. Finally, drug intervention during SE appears to lessen neuronal damage, whilst retaining epileptogenicity, suggesting that subtle alterations in neuronal function are both necessary and sufficient for epileptogenicity. In this regard, the work of Cook and Persinger [19] has shown that ‘subclincial’ low doses of lithium and pilocarpine alter spatial memory in rats and Santi et al. [30] that post-seizure treatment with ketamine prevents deficits in spatial memory. Finally, Karbowski et al [31] have recently shown that the location and degree of seizure-induced brain damage is determined by post-seizure drug treatment.

In summary, the current model shows low mortality, high morbidity, permits efficient behavioural identification of epileptic animals and shows progressive changes in electrophysiological characteristics of area CA3 without gross neuronal pathology. Some of the changes seen appear similar to those observed in human epilepsy *in vitro*. Further research will determine the usefulness of the RISE model in drug development and in the exploration of changes in other brain regions during epileptogenesis.
